# ESMO 2017—my personal highlights

**DOI:** 10.1007/s12254-018-0385-1

**Published:** 2018-02-16

**Authors:** Barbara Kiesewetter, Mircea Dediu, Rupert Bartsch

**Affiliations:** 1 Department of Medicine I, Clinical Division of Oncology, Medical University Vienna, Vienna, Austria; 2 SANADOR Hospital Bucharest, Str. Sevastopol, Nr. 9, Sector 1, 010991 Bucharest, Romania; 3 Department of Medicine I, Clinical Division of Oncology, Medical University Vienna, Vienna, Austria; 4 German Breast Group, Martin-Behaim-Straße 12, Neu Isenburg, Germany

**Keywords:** ESMO meeting, Breast cancer, Lung cancer, Lymphoma, Review

## Abstract

This article is not intended to be a comprehensive review of all highlights presented at the recent ESMO Annual Meeting, but rather a summary from a personal point of view in three very different fields of oncology. Breast cancer and lung cancer are traditionally in the focus of interest, and again, relevant new data were presented. The third part of this overview is focused on novel treatment strategies in malignant lymphoma, a field that is also quickly evolving and traditionally underrepresented at meetings dealing with solid cancers.

## Lymphoma

### (B. Kiesewetter)

Whereas orally presented original data on lymphoma were rather scarce at this year’s conference, my personal highlight was the special symposium on immunotherapy and targeted therapies held on September 9th. “I think that the true name for this is a 1000 ways of triggering T‑lymphocytes against tumor cells,” was the opening remark of chair Andrés Ferreri (Ospedale San Raffaele, Milano), underlining clearly that the development of immunomodulatory strategies has finally reached full speed also in lymphoid malignancies. This was underlined by a consistent overview on recently published data on CAR(chimeric antigen receptors)-T cells and checkpoint inhibition, discussing also novel aspects in rare lymphoma entities, e. g., pembrolizumab for primary mediastinal B‑cell lymphoma (KEYNOTE-13 study, ORR 41% in 17 r/r patients) and nivolumuab for primary central nervous system/testicular lymphoma (case series, Nayak L et al. *Blood* 2017) [1, 2]. However, these are very small cohorts and future data have to be awaited. Of public interest was the final presentation of the ASSIST-FL study, providing evidence for biosimilarity of rituximab-generic GP2013 in the first-line treatment of advanced follicular lymphoma (abstract 994O, *N* treated patients = 637) [3]. The corresponding ORR in combination with chemo backbone CVP (cyclophosphamide, vincristine, prednisolone) was 87.1% in the GP2013 arm versus 87.5% in the rituximab cohort (ORR at week 24–0.4%; 95%CI −5.94 to +5.14%). Based on the EMA approval in June 2017, the invited discussant for this abstract, Michele Ghielmini (Oncology Institute of Southern Switzerland, Bellinzona), concluded that despite the fact that long-term follow-up has to be awaited, we should trust health authorities and feel save in prescribing registered biosimilars.

## Lung cancer

### (M. Dediu)

Important advances in the lung cancer field, potentially practice changing, were communicated at the ESMO congress this year. Compared with placebo, consolidation therapy with durvalumab (a PD-L1 inhibitor) following concurrent chemoradiation in stage III NSCLC induced a significant 11-month difference in mPFS: 16.8 vs 5.6 months (HR = 0.52, *p* < 0.0001) [4]. In EGFR-mutant first-line setting, osimertinib prompted an impressive 8‑month improvement in mPFS (HR = 0.46, *p* < 0.0001) in a head-to-head comparison with first generation TKIs. Similar benefit was recorded in patients with brain metastases (HR = 0.47) [5]. Duration of nivolumab in second-line therapy should be maintained until disease progression, as treatment discontinuation at 1 year would provide a lesser survival benefit [6]. Intensive follow-up following radical surgery, using CT scan every 6 months in the first years, is no better than clinical evaluation and conventional Rx [7]. Compared with the chemotherapy alone, the benefit of combining pembrolizumab with chemotherapy in first-line setting is maintained after 18.7 months (RR = 57% vs. 32%; *P* = 0.0029; PFS 19.0 vs 8.9 months). Benefit seems to occur regardless of PD-L1 expression [8]. With an ORR of 64%, combination of dabrafenib plus trametinib appears to be a candidate for a new standard in BRAF-mutant NSCLC [9].

## Breast cancer

### (R. Bartsch)

None of the studies presented at this year’s ESMO Meeting may be deemed immediately practice changing; still, several interesting results were reported.

While well established in other malignancies, the exact role of immune checkpoint modulators is still ill defined in breast cancer. In recent months, several studies have reported a meaningful benefit in a subset of patients, but PD-L1 expression has not been convincingly shown to predict for response. Meanwhile, it is known that a high rate of tumor-infiltrating lymphocytes (TILs) is associated with better prognosis and better chemotherapy response; Loi et al. presented data suggesting that the same may be true for pembrolizumab [10]. In patients with tumors harboring high TIL infiltration and PD-L1 expression receiving immunotherapy as first-line treatment, response rate was as high as 50%.

CDK4/6 inhibitors are well established in hormone receptor-positive metastatic breast cancer (MBC). Abemaciclib is the third compound of this class and currently in advanced clinical development; now, results from the first-line MONARCH-3 study were presented [11]. Like previous data with palbociclib and ribociclib, a clinically relevant reduction of progression risk was reported (HR 0.54). These results again indicate the importance of CDK4/6 inhibitors in MBC, but the optimal treatment sequence awaits further clarification.

A French study compared chemotherapy to letrozole plus palbociclib as neoadjuvant therapy in hormone receptor-positive early breast cancer [12]. While chemotherapy yielded a higher pathologic complete response rate, response rate and breast conservation rate were comparable. While not a standard approach, these data suggest that chemotherapy-free treatment approaches should be further investigated in luminal breast cancer.

Finally, a subgroup analysis of the MINDACT trial indicated that high-risk disease as defined by gene expression can be found even among very early stage breast tumors (pT1a, pT1b, pN0) [13]. If adjuvant chemotherapy is administered in such clinically low but genomically high-risk cancer, the 5‑year disease-free survival is improved from 91.4% (95% CI 82.6–95.9) to 97.3% (95% CI 89.4–99.3), again indicating that biology is more relevant than size.
